# Liver transplantation for colorectal metastases following hepatic resection

**DOI:** 10.1093/bjs/znaf193

**Published:** 2025-09-19

**Authors:** Sheraz Yaqub, Kristoffer Watten Brudvik, Tor Magnus Smedman, Trygve Syversveen, Pål-Dag Line, Svein Dueland

**Affiliations:** Section of Hepatopancreatobiliary (HPB) Surgery, Department of Gastrointestinal and Paediatric Surgery, Oslo University Hospital, Oslo, Norway; Institute of Clinical Medicine, University of Oslo, Oslo, Norway; Section of Hepatopancreatobiliary (HPB) Surgery, Department of Gastrointestinal and Paediatric Surgery, Oslo University Hospital, Oslo, Norway; Department of Oncology, Oslo University Hospital, Oslo, Norway; Transplant Oncology Research Group, Division of Surgery and Specialized Medicine, Oslo University Hospital, Oslo, Norway; Department of Radiology and Nuclear Medicine, Oslo University Hospital, Oslo, Norway; Institute of Clinical Medicine, University of Oslo, Oslo, Norway; Transplant Oncology Research Group, Division of Surgery and Specialized Medicine, Oslo University Hospital, Oslo, Norway; Section for Transplant Surgery, Department of Transplantation Medicine, Oslo University Hospital, Oslo, Norway; Transplant Oncology Research Group, Division of Surgery and Specialized Medicine, Oslo University Hospital, Oslo, Norway

Liver resection remains the cornerstone of curative treatment for colorectal liver metastases (CRLM), yet more than half of patients experience recurrence. Repeat hepatectomy is feasible in a minority and can provide outcomes comparable to initial hepatectomy, with similar oncologic results for both early and late recurrences^1^. Liver transplantation (LT) has re-emerged as a potentially curative option for unresectable CRLM in highly selected patients^2,3^. However, comparative outcomes between LT and repeat resection for liver-limited recurrence after hepatectomy are unknown.

We performed a cohort study of patients treated with liver resection (January 2012–December 2015) or LT (December 2006–May 2020) at Oslo University Hospital, Norway. Details are presented in the Supplementary material. Patients with liver-only recurrent CRLM after initial liver resection were included; all met Oslo University Hospital LT criteria^4^. Notably, patients in the LT group were deemed unresectable by a multidisciplinary tumour board, based on the number, distribution, or location of metastatic lesions. The primary outcome was overall survival (OS), and the secondary outcomes were disease-free survival (DFS) and survival after recurrence (SAR). Outcomes were calculated from the date of second liver resection or LT using Kaplan–Meier estimates, with comparisons by the log-rank test. Categorical variables were compared using Fisher Exact test.

Ten patients underwent LT after one or more liver resections, and 43 patients underwent repeat liver resection. Baseline characteristics are shown in *Table 1*. The repeat resection group had higher age and Oslo score. The 5-year OS was significantly higher in the LT group than in the repeat resection group 90% (95% c.i., 55.5% to 99.7%) *versus* 44% (95% c.i., 29.1% to 60.1%); *P* = 0.013). Median OS was not reached in the LT group and was 54.6 months (95% c.i., 32.0 to 77.3) in the repeat resection group (*P* = 0.021) (*Fig. 1a*). Median DFS was 11.6 months (95% c.i., 0.0 to 31.5) following LT and 18.2 months (95% c.i., 12.3 to 24.1) after repeat resection (*P* = 0.511) (*Fig. 1b*).

**Fig. 1:**
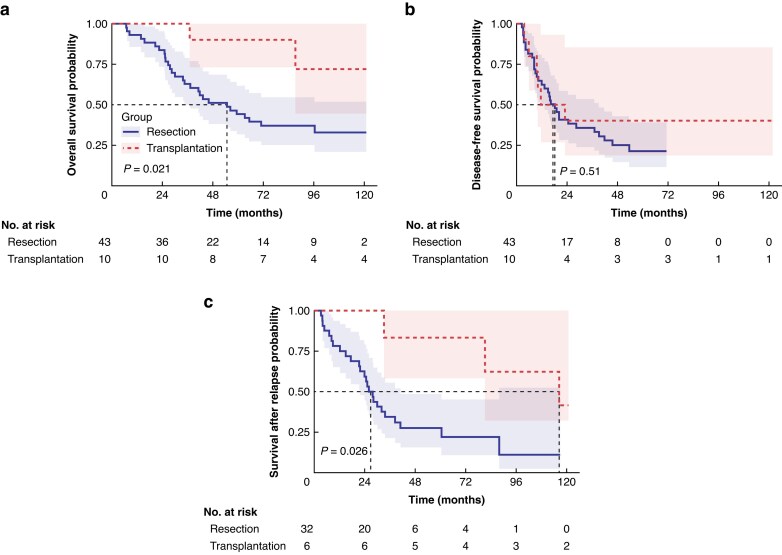
Kaplan–Meier survival curves: a overall survival after second liver-directed treatment; b disease-free survival; c survival after recurrence: liver transplant (red) *versus* repeat liver resection (blue)

**Table 1:** Baseline characteristics of patients treated with liver transplant and liver resection

Characteristic	Liver transplant	Liver resection	*P*
Patients	10	43	NA
Age, years	57.0 (51.0–61.5)	65.0 (58.7–68.0)	0.017
**Sex**			0.488*
Male	4	24	
Female	6	19	
**Primary tumour location**			
Right colon	0	7	
Transverse colon	1	0	0.899
Left colon	4	17	
Rectum	5	19	
**AJCC TNM stage**			
T1	0	1	0.729
T2	3	7	
T3	6	26	
T4	1	7	
Unknown T-stage	0	2	
N0	5	18	0.713
N1	3	16	
N2	2	9	
**KRAS status**			
Wild-type	9	15	0.148
Mutated	1	11	
Unknown	0	17	
CEA†, µg/l	2.2 (1.0–4.8)	3.1 (1.8–5.4)‡	0.777
Largest metastasis§, mm	14.0 (10.0–28.3)	15.0 (12.0–23.0)	0.776
No. of metastases§	3 (2–4)	2 (1–3)	0.161
Oslo score¶	0 (0–0)	1 (0–1)	0.003*
**Site of recurrence**			
Lungs	6	9	0.005
Liver	0	12	
Multiple sites	0	11	

Data are presented *n* or median (i.q.r.) unless otherwise indicated. Groups were compared using the Mann–Whitney U test unless otherwise specified. CEA, carcinoembryonic antigen; NA, not applicable. *Two-sided Fisher Exact test. †Measured at time of second liver resection or liver transplantation. ‡Values missing for 2 patients. §Measured on preoperative CT or MRI of the liver. ¶Oslo score assigns 1 point for each of the following: CEA > 80 µg/l, progressive disease on chemotherapy, largest tumour diameter >55 mm, or time from diagnosis to transplant <2 years.

Recurrence occurred in 6 of 10 LT patients (median SAR, 116.6 months; 95% c.i., 42.8 to 190.4) and in 32 of 43 resection patients (median SAR, 26.1 months; 95% c.i., 21.4 to 30.7; *P* = 0.026) (*Fig. 1c*). Recurrences after LT were exclusively pulmonary, whereas recurrences after repeat resection were mainly intrahepatic or multiple extrahepatic sites (*Table 1*). Among LT patients with recurrence, 5 of 6 underwent lung resection. In the resection group, patients received curative-intent treatment for lung (5 of 9), liver (10 of 12), or multiple site (1 of 11) metastases. There was no 90-day postoperative death in either group.

## Supplementary Material

znaf193_Supplementary_Data

## Data Availability

Owing to restrictions on participant consent and data-sharing policies, the supporting data are not publically available.

## Data sharing statement

Owing to restrictions on participant consent and data-sharing policies, the supporting data are not publicly available.
