# Supplementary material for: Liver transplantation for colorectal metastases following hepatic resection
Source: Br J Surg. 2025 Sep 19;112(9):znaf193. doi: 10.1093/bjs/znaf193 (PMC12449174; doi:10.1093/bjs/znaf193)
Supplement: znaf193_Supplementary_Data [file znaf193_supplementary_data.docx]

**Liver transplantation for colorectal metastases following hepatic resection**

Sheraz Yaqub^1,2^, Kristoffer Watten Brudvik^1^, Tor Magnus Smedman^3,4^, Trygve Syversveen^5^, Pål-Dag Line^2,4,6^, Svein Dueland^4^

1. Section of Hepato-pancreato-biliary (HPB) Surgery, Department of Gastrointestinal and Paediatric Surgery, Oslo University Hospital, Oslo, Norway.
2. Institute of Clinical Medicine, University of Oslo, Oslo, Norway.
3. Department of Oncology, Oslo University Hospital, Oslo, Norway.
4. Transplant Oncology Research Group, Division of Surgery and Specialized Medicine, Oslo University Hospital, Oslo, Norway.
5. Department of Radiology and Nuclear Medicine, Oslo University Hospital, Oslo, Norway.
6. Section for Transplant Surgery, Department of Transplantation Medicine, Oslo University Hospital, Oslo, Norway.

**Corresponding author:**

Sheraz Yaqub, MD PhD FEBS

Department of Hepatopancreatobiliary Surgery

Oslo University Hospital

Oslo N-0372

Norway

E-mail: [sheraz.yaqub@medisin.uio.no](mailto:sheraz.yaqub@medisin.uio.no)

Twitter/X: @yaqubsheraz

ORCID: <https://orcid.org/0000-0002-5696-2319>

**Supplementary Material - Index**

| **Supplementary Methods** | *page 3* |
| --- | --- |
| Ethical consideration | *Page 3* |
| **Supplemental Appendixes** | *page 3* |
| Discussion and Limitations | *page 3* |
| Conclusion | *page 3* |
| **References** | *page 4* |

**Supplementary Methods**

*Ethical consideration*

All patients with previous history of liver resection for colorectal liver metastases (CRLM) who underwent liver transplantation (LT) between December 2006 and May 2020 at Oslo University Hospital, Norway, were part of prospective approved clinical studies (SECA-I^1^ and SECA-II^2^ [clinicaltrials.gov #: NCT01311453 and NCT01479608]). All protocols were approved by the Regional Committee for Medical and Health Research Ethics and the Institutional Review Board of Oslo University Hospital. All patients provided written informed consent before inclusion, which was obtained in a manner that was consistent with the Declaration of Helsinki. No financial compensation or incentives were provided to patients for their participation in the studies.

**Supplementary Appendixes**

*Discussion and Limitations*

In this comparative analysis of patients with liver-only recurrent CRLM after prior hepatectomy, LT was associated with significantly improved long-term overall survival (OS) and survival after recurrence (SAR) compared with repeat resection. This survival benefit was observed despite the application of similar transplant eligibility criteria across the study population. In contrast to patients undergoing a repeat liver resection, all patients undergoing LT had been deemed non-resectable by a multidisciplinary tumour board, based on the number, distribution, or location of metastatic lesions. Furthermore, patients undergoing repeat liver resection had a higher median Oslo score, indicating a higher cumulative burden of adverse prognostic factors.^3^

While previous studies have supported the feasibility and survival benefit of LT for selected patients with CRLM,^4^ this study represents, to our knowledge, the first direct comparison of outcomes between LT and repeat resection specifically in patients with prior hepatic surgery. The superior OS observed in the LT cohort likely reflects the unique capacity of liver transplantation to address both macroscopic and occult hepatic disease, particularly in the setting of chemotherapy-altered or surgically modified liver parenchyma, where the efficacy of repeat resection may be inherently limited.

Study strengths include standardized eligibility criteria for LT for both groups and extended duration of follow-up, allowing for mature survival and recurrence data. However, several limitations warrant consideration. These include the retrospective nature of the analysis, the small sample size, especially in the LT cohort, and potential selection bias. Specifically, the LT group may have included patients who, due to being selected for multiple resections prior to transplant, inherently had more indolent or liver-confined disease biology. This selection bias may also contribute to the higher observed rate of extrahepatic recurrence in the resection cohort, reflecting a potential for more aggressive disease spread in that group.

*Conclusion*

In summary, LT was associated with markedly improved long-term OS and SAR compared with repeat resection in patients with liver-only recurrent CRLM after hepatectomy. These findings suggest LT should be considered earlier for selected patients with recurrent, liver-only CRLM.

**References**

1. Hagness M, Foss A, Line PD, Scholz T, Jorgensen PF, Fosby B, et al. Liver transplantation for nonresectable liver metastases from colorectal cancer. *Ann Surg* 2013;257:800-806

2. Dueland S, Syversveen T, Solheim JM, Solberg S, Grut H, Bjornbeth BA, et al. Survival Following Liver Transplantation for Patients With Nonresectable Liver-only Colorectal Metastases. *Ann Surg* 2020;271:212-218

3. Dueland S, Smedman TM, Syversveen T, Grut H, Hagness M, Line PD. Long-Term Survival, Prognostic Factors, and Selection of Patients With Colorectal Cancer for Liver Transplant: A Nonrandomized Controlled Trial. *JAMA Surg* 2023;158:e232932

4. Adam R, Piedvache C, Chiche L, Adam JP, Salame E, Bucur P, et al. Liver transplantation plus chemotherapy versus chemotherapy alone in patients with permanently unresectable colorectal liver metastases (TransMet): results from a multicentre, open-label, prospective, randomised controlled trial. *Lancet* 2024;404:1107-1118
